# Building predictive disease models using extracellular vesicle microscale flow cytometry and machine learning

**DOI:** 10.1002/1878-0261.13362

**Published:** 2022-12-29

**Authors:** Robert J. Paproski, Desmond Pink, Deborah L. Sosnowski, Catalina Vasquez, John D. Lewis

**Affiliations:** ^1^ Department of Oncology University of Alberta Edmonton AB Canada; ^2^ Nanostics Inc. Edmonton AB Canada

**Keywords:** cancer prediction, diagnostic test, extracellular vesicles, machine learning, microflow cytometry, prostate cancer

## Abstract

Extracellular vesicles (EVs) are highly abundant in human biofluids, containing a repertoire of macromolecules and biomarkers representative of the tissue of origin. EVs released by tumours can communicate key signals both locally and to distant sites to promote growth and survival or impact invasive and metastatic progression. Microscale flow cytometry of circulating EVs is an emerging technology that is a promising alternative to biopsy for disease diagnosis. However, biofluid‐derived EVs are highly heterogeneous in size and composition, making their analysis complex. To address this, we developed a machine learning approach combined with EV microscale cytometry using tissue‐ and disease‐specific biomarkers to generate predictive models. We demonstrate the utility of this novel extracellular vesicle machine learning analysis platform (EVMAP) to predict disease from patient samples by developing a blood test to identify high‐grade prostate cancer and validate its performance in a prospective 215 patient cohort. Models generated using the EVMAP approach significantly improved the prediction of high‐risk prostate cancer, highlighting the clinical utility of this diagnostic platform for improved cancer prediction from a blood test.

## Graphical abstract

We developed the extracellular vesicle machine learning analysis platform (EVMAP) to improve the prediction of diseases such as cancer. The platform combines extracellular vesicle analysis using microscale cytometry with a machine learning approach to generate predictive models. In this work, we utilized EVMAP to generate a blood test to predict high‐grade prostate cancer in men that was significantly more accurate than the prostate‐specific antigen test. This platform could be applied to many different diseases.

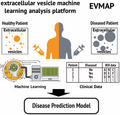

**Abbreviations**
APCaRI: Alberta Prostate Cancer Research Initiative
Deep CNN: Deep Convolutional Neural Network
DRE: digital rectal exam
EV: extracellular vesicle
EVMAP: extracellular vesicle machine learning analysis platform
FCS: flow cytometry standard
LALS: large angle light scatter
PSA: prostate‐specific antigen
PSMA: prostate‐specific membrane antigen
RI: refractive index
ROC AUC: receiver operator characteristic area under the curve
ROI: regions of interest
SOC: standard‐of‐care
XGBoost: Extreme Gradient Boosting
μFCM: microflow cytometry

## 1 Introduction

Cells continuously release extracellular vesicles (EVs) such as exosomes (30–100 nm), microvesicles (50–2000 nm), apoptotic bodies (500–4000 nm) and oncosomes (1000–10 000 nm) that contain abundant mRNA, miRNA and protein biomarkers from their cells of origin for intercellular communication [1, 2]. EVs from diseased cells display disease‐specific biomarkers and may be found in biofluids, including blood, breast milk, cerebrospinal fluid, saliva, semen, sweat and urine [3, 4]. Therefore, EVs represent a rich pool of physiological disease‐state biomarkers that can be leveraged to develop minimally invasive diagnostic and prognostic assays [5, 6, 7, 8].

Characterization methods for EVs vary in resolution, speed and ease of analysis [9]. Electron microscopy can provide high‐resolution EV images but requires complicated image analysis, and it cannot easily measure multiple biomarkers simultaneously, making this a low‐throughput approach [10]. Nanoparticle tracking analysis and tuneable elastomeric pulse sensors allow rapid enumeration and sizing of nano‐ and micro‐particles but are not ideal for characterizing biomarkers that are associated with EVs [11, 12]. Conversely, microscale flow cytometry or microflow cytometry (μFCM), a high‐resolution form of flow cytometry, allows high‐throughput characterization of millions of nanometre‐sized EVs within minutes, including estimation of particle size by light scatter, concentration and marker abundance via fluorescence [9, 13, 14].

Microflow cytometry analysis of EVs from patient biofluids is complex due to the substantial intra‐ and inter‐patient heterogeneity in the EV population and the potential unknown of which EV population(s) provides the most clinical value. Traditional flow cytometry analysis methods are not optimal to analyse μFCM data of EVs since they focus on events in a relatively narrow micrometre size range for cells. Therefore, improved analysis methods are required to maximize the diagnostic and prognostic value of clinical μFCM data.

Prostate cancer is the most diagnosed cancer in men and the second leading cause of male cancer deaths in the US [15, 16]. Most men are diagnosed with low‐grade, localized prostate cancer and have a 5‐year net survival rate of 100%. However, the 5‐year survival rate drops to 32% for men diagnosed with high‐grade (grade group (GG) ≥ 3), metastatic prostate cancer [17, 18]. Current diagnostic tests, such as the prostate‐specific antigen (PSA) blood test, lack specificity for predicting grade group (GG) ≥ 3 prostate cancer. Therefore, there is an unmet clinical need for minimally invasive tests to improve the detection of GG ≥ 3 prostate cancer.

Here, we describe the development of a novel predictive extracellular vesicle machine learning analysis platform (EVMAP) to generate disease‐specific scores from μFCM and clinical data using a purpose‐built machine learning approach. We apply EVMAP to the development of a blood test to predict the likelihood of high‐grade prostate cancer in men with elevated PSA that have been referred for prostate biopsy. The test utilizes μFCM to assess the concentration of two biomarkers (PSMA and ghrelin) [19, 20, 21] on plasma‐derived EVs combined with six associated clinical features. We found that the EVMAP approach was significantly more accurate compared to gating the EV biomarker data manually. While manual gating resulted in a receiver operator characteristic area under the curve (ROC AUC) of 0.52, EVMAP resulted in an AUC of 0.75 for the prediction of (GG) ≥ 3 prostate cancer. This EVMAP approach could easily be adapted to predict a number of other high‐grade cancers and diseases.

## 2 Materials and methods

### 2.1 Patient characteristics and sample acquisition

Pre‐biopsy plasma samples from 215 men suspected of prostate cancer were acquired from the Alberta Prostate Cancer Research Initiative (APCaRI) biorepository [22]. The clinical study was approved by the Health Research Ethics Board of Alberta under the APCaRI‐01 protocol (HREBA‐CC‐18‐0513). The study methodologies conformed to the standards set by the Declaration of Helsinki. The inclusion criteria were adult men without prior prostate cancer diagnosis: (a) referred to urology clinics in Alberta for prostate concerns and scheduled for a prostate biopsy; or (b) undergoing transurethral prostate surgery for diagnosis or treatment of prostate abnormalities. All patients provided written informed consent, and the scientific ethics committees approved the study at the Prostate Cancer Centre (Calgary, AB, Canada) and the Kipnes Urology Centre (Edmonton, AB, Canada). Patients were enrolled in the study between June 2014 and September 2015. Transrectal ultrasound‐guided prostate biopsies were performed with a median of 12 cores per patient and evaluated according to each hospital's standard operating procedure (SOP). Test results were not provided to the clinical sites for patient care. Laboratory personnel who acquired patient samples and ran tests were blinded for patient characteristics. Blood was collected and processed to collect plasma as per institutional SOP, and the time from arm to −80 °C freezer was 2 h or less.

### 2.2 Instrument set‐up and optimization

Experiments were conducted to first define the appropriate range for sample acquisition. Plasma samples were serially diluted and assayed to monitor events per second versus total median intensity (large angle light scatter, LALS). The proportion of the data that provided a ‘flat’ range, or single particle analysis, was 400–20 000 events per second. A general dilution of 100× permitted the majority of plasma samples to fall within this range of 400–20 000 events per second. Samples outside of this range were not included. Analysis of the fluorescence (FL) signal within this range showed that all biomarker signals were suitable for use. Additional studies have shown that even if the events per second rate is increased to near 100 000 events per second using the addition of a spiked negative control, the detection of an FL signal is maintained at a constant concentration and median fluorescent intensity (MFI), while the per cent positive decreases dramatically.

### 2.3 Microflow cytometry assay

Frozen plasma samples for each of the 215 patients were thawed, centrifuged at 16 000 ***g*** for 30 min to remove large debris and platelet particles, and 10 μL of sample were mixed with 3–5 μL of each antibody, reacted and finally diluted to 1 mL in low protein‐binding Eppendorf tubes (Axygen, 1.7 mL ultraclear microtubes, MCT‐175‐X; Thomas Scientific, Swedesboro, NJ, USA). Aliquots (250 μL) were added to individual wells of microtitre plates (96 well suspension culture plant, sterile, u‐bottom, with lid, Cellstar Cat# 650185; Millipore Sigma, Burlington, MA, USA). This mixture included 5 μg·mL^−1^ mouse anti‐PSMA antibody (J591, no lot number, provided by N. Bander) and a 1 : 50 dilution of secondary Qdot565‐conjugated donkey anti‐mouse IgG antibody (QDot Secondary product #Q11032MP Lot #1611101; Thermo Fisher Scientific, Waltham, MA, USA) and 0.133 mm Cy5.5‐ghrelin probe containing the first 18 amino acids of ghrelin (provided by L. Luyt). Thirty minutes after probe incubation, the samples were diluted 100‐fold in double‐filtered (0.1 μm) phosphate‐buffered saline and analysed with the Apogee A50 microflow cytometer (Apogee Flow Systems, Northwood, UK) using a flow rate of 3.01 μL·min^−1^. Events were collected for 2 min. Sample dilution at 100× was appropriate for most samples, providing an event rate of 400–20 000 events per second. This event rate was defined in prior experiments on the instrument to be suitable for analysis, no apparent coincidence. Samples outside of this range were either diluted further or excluded from analysis. Plasma from each patient was run in triplicate. Conventional manual gating analysis of μFCM data was performed using histogram version 255.0.0.80 software (Apogee Flow Systems). See Table S1 for microflow cytometry settings and Table S2 for MIFlowCyt/MISEV compliant items [23].

### 2.4 Size estimation of EVs using microflow cytometry

We are not attributing any diagnostic or prognostic potential to any vesicles of a particular size or subpopulation. While standardization of light scatter intensity to biological vesicle size would be critical for reproducibility, here we state that the detected vesicles were always within the range of the light scattering intensities of the monitoring bead mix [24, 25]. The Apogee 1493 bead mix contained two polystyrene beads 110 and 500 nm (refractive index (RI) 1.59) and six silica beads (180–1300 nm, RI 1.46) that spans the range of detected EVs in this study. EVs were detected in the range of intensities relative to the intensity of 110 nm polystyrene (lower range cut‐off) and 500 nm polystyrene (upper range cut‐off, Figs S1–S3).

### 2.5 Processing microflow cytometry data

Manual gating of FCS files was performed using a fixed square gating strategy for each scatter plot (e.g. LALS‐PSMA, LALS‐Ghrelin, PSMA‐Ghrelin) based on manual review of FCS files. This strategy was chosen for ease of bulk, consistent analysis of all FCS files. Gates on scatter plots with LALS on the *X*‐axis included particles for all values of LALS. Gates for PSMA or ghrelin started at probe signals which were slightly above the large negative population of particles found in all samples and included particles with maximum probe signal intensities.

FCS files were also analysed using a custom matlab (version R2017a) script (MathWorks, Natick, MA, USA). Within each file, signal intensities for all channels were log‐transformed, and particles with similar optical properties were binned using 32‐bins per optical channel unless stated otherwise. Three different bivariate histograms of particle concentration were created: (a) LALS and PSMA stain intensity, (b) LALS and ghrelin probe stain intensity and (c) PSMA and ghrelin probe stain intensity. Each bivariate histogram contained 1024 ROIs (32 × 32 bins). Particle concentration in each ROI was averaged over the three replicates per patient.

### 2.6 Predicting and correlating clinical features with microflow cytometry data

The μFCM data were used to: (a) predict binary clinical features (e.g. patients with or without perineural invasion, normal or abnormal digital rectal exam) and (b) correlate with ordinal or interval clinical features (e.g. tumour stage or PSA respectively) using a custom matlab script (MathWorks). An Excel instruction file describing how to analyse the μFCM data for each clinical feature was created to minimize the code needed for automated analysis. Each clinical feature within the instruction file was a separate column, and each row contained specific information or instructions. Specific information included the location of the clinical feature within the database, the type of data for each clinical feature (binary or ordinal/interval), and the value which represents missing data for that clinical feature. Instructions primarily involved how to transform the clinical feature, which included thresholding values when binarizing features, deriving the prostate cancer grade groups from Gleason scores, and determining age from date of birth. Patients missing specific clinical feature data were removed from analysis for that clinical feature.

Once clinical feature data were retrieved from the database for all patients and transformed, μFCM particle concentration data for each ROI was used to predict or correlate with clinical features. For binary clinical features, ROC area under the curve (AUC) values were determined for each ROI, and AUC maps were generated for each bivariate data set, including LALS‐PSMA, LALS‐ghrelin and PSMA‐ghrelin. For ordinal/interval clinical features, Pearson correlation coefficients were determined for each ROI, and correlation maps were generated for each bivariate data set. The highest 10% of AUC values in each AUC map were averaged, and these values were compared across clinical features.

### 2.7 viSNE analysis of microflow cytometry data

viSNE plots were created using cyt version 2.0 software run on matlab (MathWorks) [26]. Each patient's triplicate FCS files were concatenated into one file. Two new FCS files were created: one using events from patients with grade group 2 and lower prostate cancer, and the other using events from patients with grade group 3 and higher prostate cancer. These two files totalled approximately 100 000 events, with an equal number of events from each patient within their group. With cyt software, 30 000 events from both files were randomly subsampled and merged to create 60 000 events. The events were visualized with viSNE using the bh‐SNE transformation using LALS, PSMA and ghrelin channels, then clustered with the k‐means and expectation–maximization Gaussian mixture model algorithms. The viSNE results were exported from Cyt and clustered using the fast search/density peaks algorithm using the DensityClust function for matlab (MathWorks) [27]. Event pair Euclidean distances were determined using the pdist2 function. For setting delta and rho parameters using the paraSet function, the per cent neighbour variable was set to 2%, and a Gaussian kernel was used. Cluster centres were selected using delta values between 1.5 and 5 and rho values between 200 and 1900. For all clustering algorithms, 248 clusters were created over the 60 000 events. Cluster purity for high‐grade prostate cancer was defined as the number of high‐grade prostate cancer events divided by the total number of events within each cluster. Only clusters with at least 60 particles (0.1% of total particles) were analysed.

### 2.8 Optimizing machine learning models for predicting high‐grade prostate cancer

matlab's classification learner app was used to test 23 different machine learning algorithms to predict GG ≥ 3 prostate cancer using particle concentration μFCM data (MathWorks). These algorithms included individual/bagged/boosted decision trees, linear/quadratic/cubic/Gaussian support vector machines, logistic regression, linear/quadratic/subspace discriminant analysis and k‐nearest neighbours. Extreme Gradient Boosting (XGBoost) was also tested using the ‘XGBoost’ package in r (version 3.3.3) [28]. All machine learning algorithms used default settings and five‐fold cross‐validation repeated at least 10 times with patient randomization within folds for each cross‐validation repeat. Five‐fold cross‐validation divides the data into five groups and, in the first iteration, trains a model with four of five groups (i.e. 80% of the data) and evaluates the model on the group not used for training (i.e. 20% of the data). The process repeats four more times and changes the group used for model evaluation for each iteration until all data are used for validation once. The predictions made on the held‐out group (i.e. not used for model training) were used for determining model performance using the AUC.

The machine learning algorithm with the highest AUC was then optimized by (a) comparing 2, 4, 8, 16, 32, 64 and 128 bins when processing the μFCM data, (b) creating ensembles of 3, 6, 12, 25, 50 and 100 models using the same machine learning algorithm but randomly selecting different subsets of patients as training data and averaging model predictions, (c) selecting the best subset of μFCM ROIs using recursive feature elimination with the r ‘caret’ package, and (d) grid searching algorithm parameters (XGBoost: nrounds = 50, 100, 150, 200, 250, 300, 400; max_depth = 3, 4, 5, 6; eta = 0.01, 0.1; gamma = 0; colsample_bytree = 1; min_child_weight = 1; subsample = 1). The binning/ensembling/features/parameters that provided the highest AUCs were used together to create a final model for predicting high‐grade prostate cancer. This model was compared to manual gating analysis using histogram software, and CITRUS with default settings using r. CITRUS predicts clinical conditions from flow cytometry data by using hierarchical clustering and lasso‐regularized logistic regression and nearest shrunken centroid methods [29].

A logistic regression model was created using SOC clinical features, which included PSA, age, DRE, family history of prostate cancer, previous negative biopsy and race (black = 1, other races = 0) and the final μFCM model probability predictions. This model was compared to a similar logistic regression model without using μFCM data.

### 2.9 Statistical analysis

Unless stated otherwise, bar/dot plots with error bars represent mean ± standard error of the mean. When comparing two groups, unpaired two‐tailed *t*‐tests were used for interval data, and Fisher's exact tests were used for binary categorical variables. One‐way ANOVA was used for comparing three or more groups using Tukey's multiple comparison test. ROC curves were compared by DeLong's method using the ‘pROC’ package in r. When possible, ROC cut‐off values were determined using ~ 90% sensitivity, and the resulting specificity and positive/negative predictive values were determined using graphpad prism version 6.01 software (GraphPad Software, San Diego, CA, USA).

## 3 Results

To develop and evaluate EVMAP, μFCM data were collected from plasma samples from 215 men at risk of prostate cancer with the goal of predicting the subset of men with prostate cancer. Patient characteristics for the 215‐patient cohort are described in Table S3.

### 3.1 PSMA and ghrelin microflow cytometry data best predict (GG) ≥ 3 prostate cancer

**Fig. 1:**
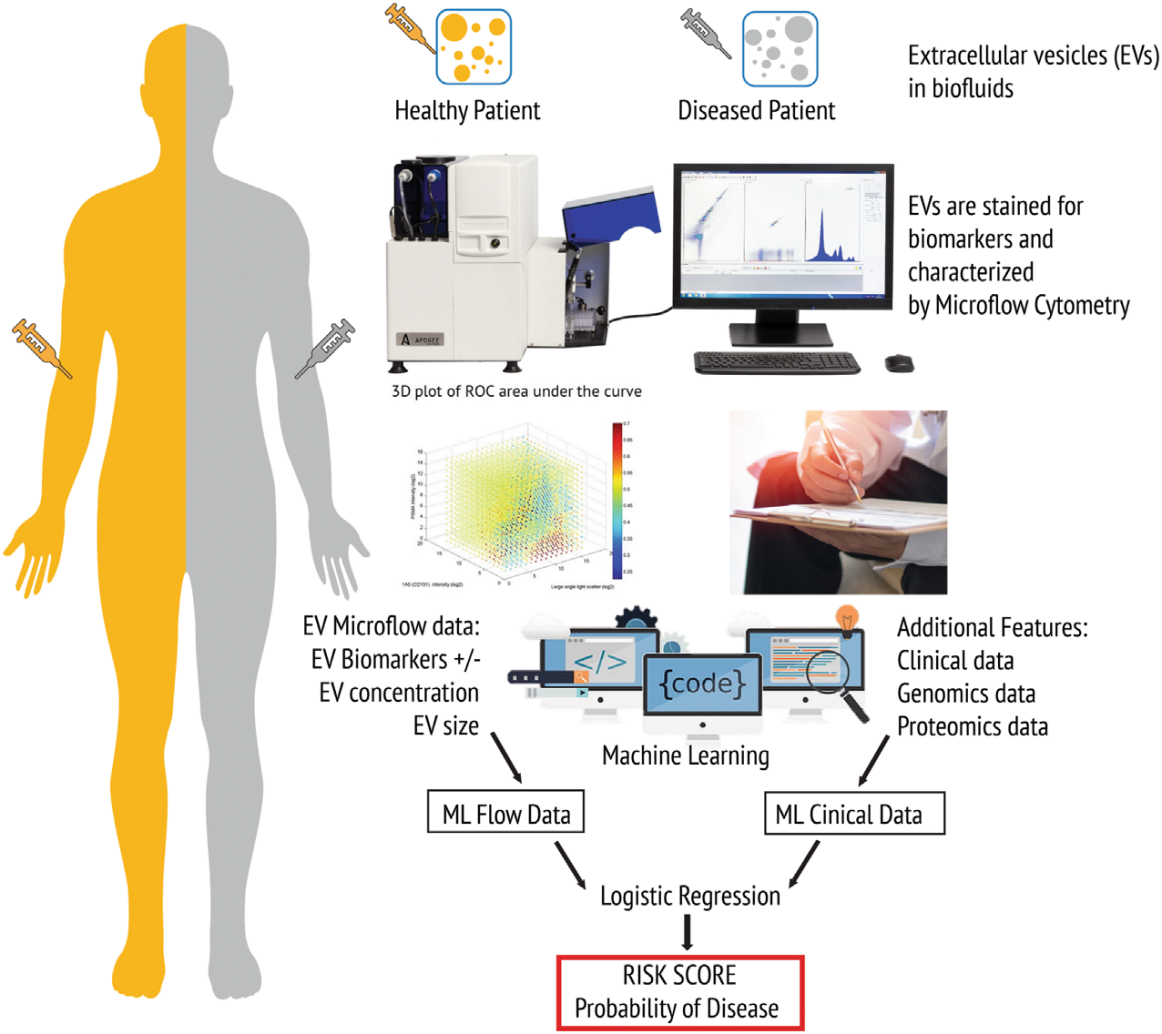
Flow diagram of the extracellular vesicle (EV) machine learning platform (EVMAP) technology. Healthy and diseased cells secrete a heterogenous population of EVs into the surrounding biofluid. The EV population in the patient sample is stained with disease‐specific biomarkers then the EV fluorescence, EV concentrations and EV sizes are quantified by microflow cytometry. The resulting flow data and the patient clinical data are then analysed by machine learning to generate prediction models from both the flow data and clinical data which is further analysed by logistic regression to determine the patient's risk score indicating probability of disease.

Since different EV populations have different origins and sizes, there will be substantial variation in their predictive value for disease states. For this reason, we divided the μFCM data into regions of interest (ROIs) where the concentrations of EVs could be precisely determined and correlated with clinical conditions (Fig. 1). Each microflow cytometer channel (e.g. LALS, PSMA signal) was divided into 32 bins which created 1024 ROIs for each two‐dimensional scatter plot. The concentration of particles in each ROI was used to predict disease states and an AUC was calculated for each ROI. Using this approach, we generated AUC maps of the μFCM data for each clinical condition available in the patient data set, emphasizing conditions relevant to high‐grade prostate cancer diagnosis.

**Fig. 2:**
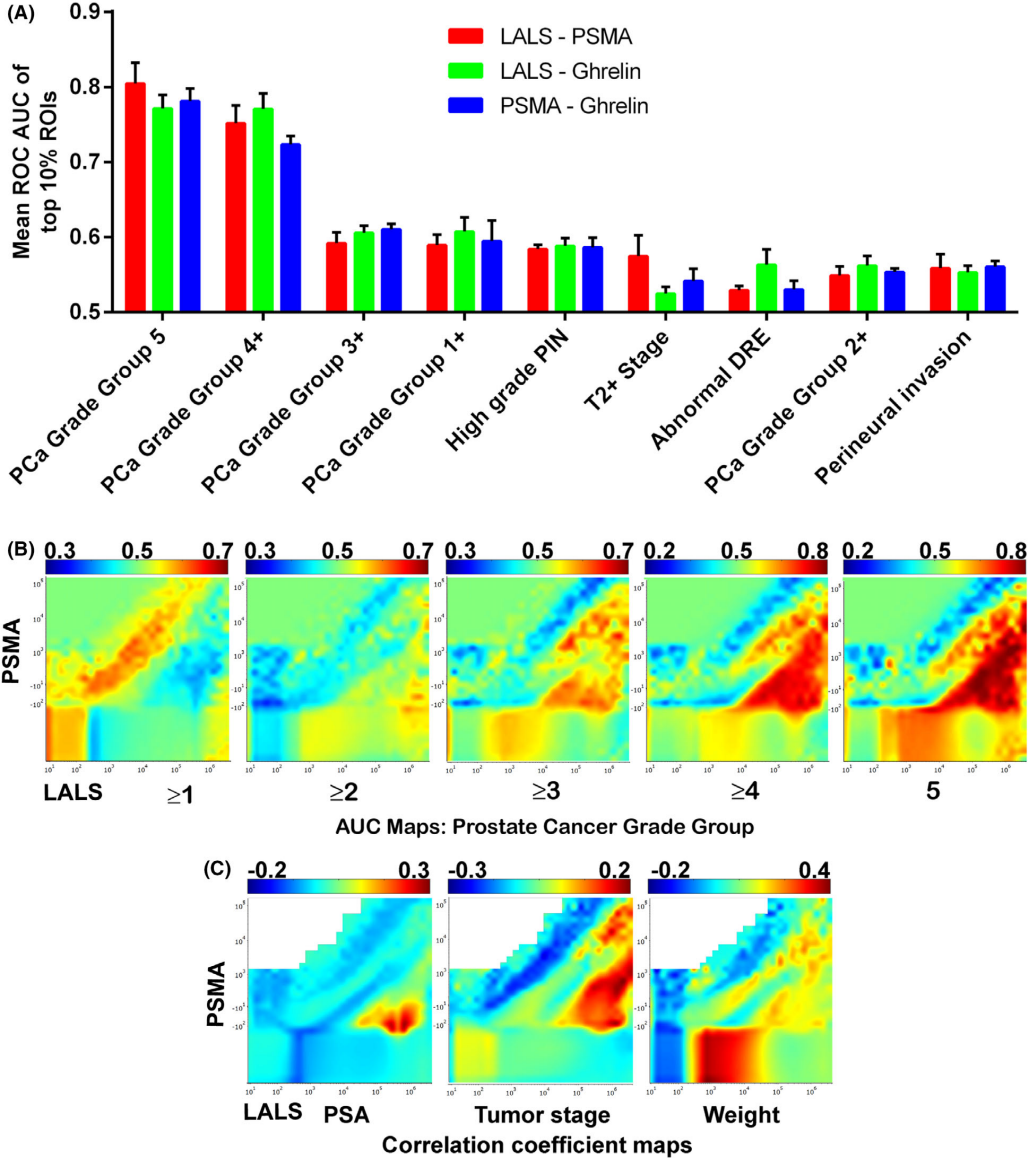
The extracellular vesicle (EV) machine learning platform (EVMAP) uses microflow cytometry receiver operator characteristic area under the curve (ROC AUC) maps to identify the clinical state the EV biomarker data best predicts. (A) ROC AUC maps for predicting clinical features using the large angle light scatter‐prostate‐specific membrane antigen (LALS‐PSMA), LALS‐ghrelin and PSMA‐ghrelin data sets. The highest 10% AUCs in each map were averaged and compared. Error bars indicate standard deviation. (B) ROC AUC maps for predicting prostate cancer grade group ≥ 1, ≥ 2, ≥ 3, ≥ 4, and 5 using the LALS‐PSMA data set. (C) Correlation coefficient maps for prostate‐specific antigen (PSA; left), tumour stage (middle), and weight (right) using the LALS‐PSMA data set.

When averaging the top 10% of AUCs within the LALS‐PSMA, LALS‐ghrelin and PSMA‐ghrelin AUC maps, predicting prostate cancer grade group 5 and ≥ 4 provided the highest averaged AUCs (Fig. 2A). Notably, all three bivariate AUC maps provided top 10% AUCs above 0.7 for predicting high‐grade prostate cancer, with LALS‐PSMA having an AUC above 0.8 for predicting grade group 5 prostate cancer. The LALS‐PSMA AUC maps displayed a consistent particle distribution shift associated with increasing prostate cancer grade group (Fig. 2B). When estimating particle size using LALS, prediction of grade group ≥ 1 displayed relatively smaller PSMA‐positive particles with AUCs above 0.5. Therefore, particle concentration in these ROIs was generally higher in patients with grade group ≥ 1 prostate cancer. Conversely, larger PSMA‐positive particles mostly displayed AUCs below 0.5, so particle concentration in these ROIs was generally lower in patients with grade group ≥ 1 prostate cancer. The AUC maps for higher grade groups demonstrated a progressive inversion of this phenotype, with grade group 5 having AUCs > 0.8 for larger PSMA‐positive particles and AUCs approximately 0.3 for many smaller PSMA‐positive particles. This phenotype inversion was quite noticeable with grade group ≥ 3 AUC maps. The greater abundance of larger PSMA‐positive particles in higher‐grade prostate cancer patients may be partly due to circulating metastatic cells [30]. Further experiments are necessary to provide evidence for this hypothesis.

We used the LALS‐PSMA data to create correlation maps for the PSA levels, tumour stage and weight (Fig. 2C). Large weakly positive PSMA particles demonstrated the highest positive correlation with PSA, whereas large PSMA‐positive particles correlated best with the tumour stage. These outcomes may be due to the correlation of prostate PSMA and PSA at diagnosis [31], and higher‐grade tumours are more likely to spread, potentially explaining the similarity between the higher‐grade AUC maps and the tumour stage correlation map. Interestingly, small PSMA‐negative particles showed a relatively strong positive correlation for weight, although the identity of these particles remains unknown.

Given the results of the AUC‐correlation maps, we focused on utilizing the μFCM data to predict high‐grade high‐risk prostate cancer, which we defined as grade group ≥ 3 since these patients demonstrate significantly worse outcomes than grade group 2 and lower prostate cancer patients [32].

### 3.2 Manual gating of microflow cytometry data results in poor predictive performance

**Fig. 3:**
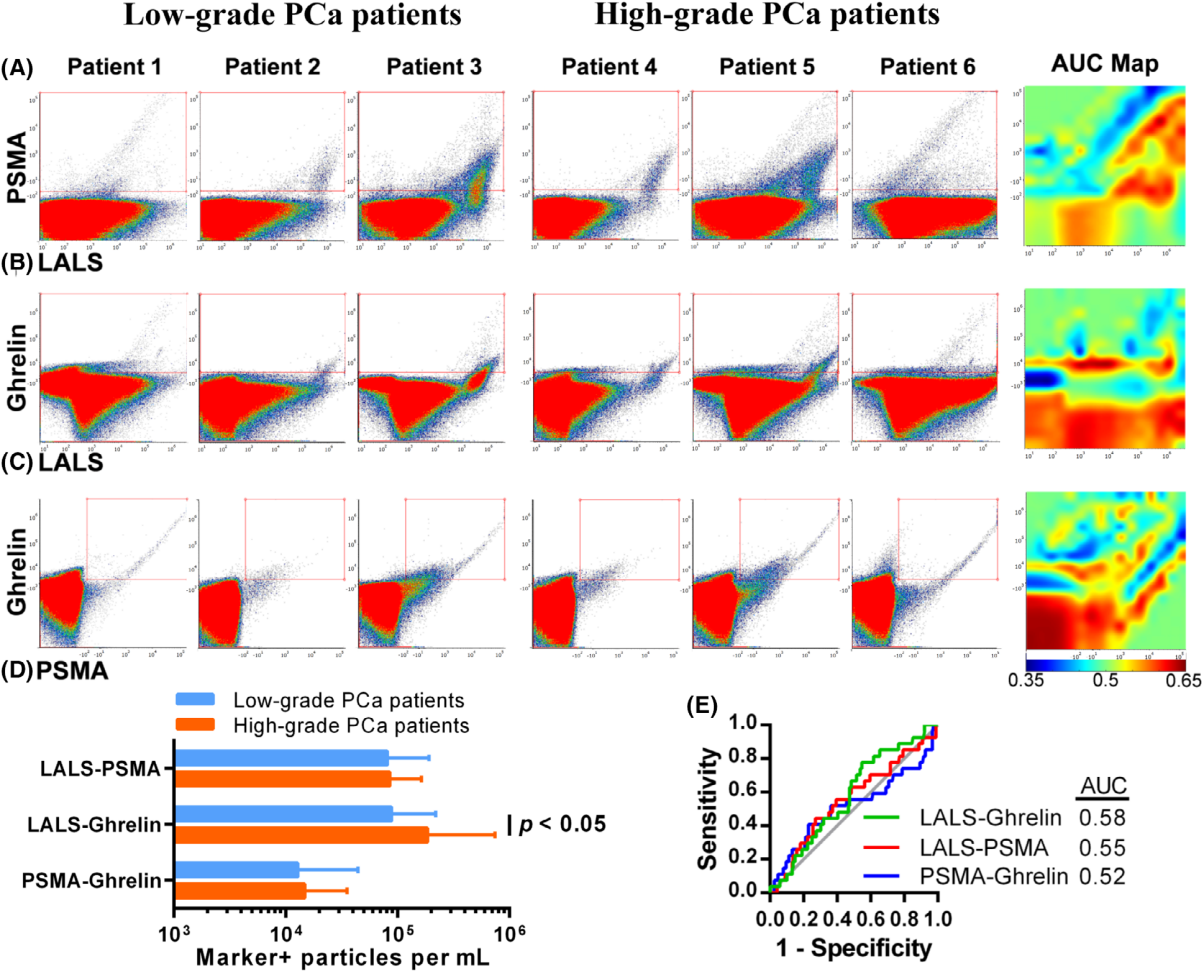
Variability of prostate‐specific membrane antigen (PSMA)/ghrelin probe particle staining from plasma samples complicates conventional manual gating analysis. Representative scatter plots and receiver operator characteristic (ROC) area under the curve maps of: (A) Large angle light scatter (LALS) and PSMA, (B) LALS and ghrelin, and (C) PSMA and ghrelin for patients with low‐grade and high‐grade prostate cancer. (D) Quantitation of PSMA/ghrelin probe positive particles in patient plasma by manual gating. Error bars indicate standard deviation. Statistical analysis was done by two‐tailed unpaired *t*‐test in panels B and D. (E) ROC curves for predicting high‐grade prostate cancer using manual region of interest data.

We first evaluated the quantification of μFCM data using manual gating to assess whether a simplistic analysis could provide adequate predictive power. Applying manual gates around specific particle populations is a non‐trivial task due to the high complexity of the multi‐parametric data (Fig. 3A–C). For simplicity, we created gates that grouped all marker‐positive particles. When compared to low‐grade prostate cancer, only the concentration of ghrelin‐positive particles was significantly higher in high‐grade prostate cancer by 2.1‐fold (*P* < 0.05) (Fig. 3D). The AUCs of PSMA‐, ghrelin‐ and PSMA/ghrelin‐positive particle concentrations for predicting high‐grade prostate cancer were all below 0.6 (Fig. 3E). These low AUCs are easily explained by the AUC maps, demonstrating that simple gating will group particles with high and low AUCs (Fig. 3A–C). It was clear that the high dimensionality of the data and the heterogeneity in EV particle sizes necessitated a more sophisticated gating approach.

We mapped the high‐dimensional μFCM data with the viSNE algorithm, which allowed the conservation of the high‐dimensional structure of the data into an easily visualized two‐dimensional format [26]. viSNE maps are also useful for identifying rare cell populations [33]. The viSNE plot, generated using an equal number of particles from high‐grade and low‐grade prostate cancer patients, revealed more particle populations than were apparent with conventional scatter plots (Figs S4 and S5A). Particles were clustered using K‐means, expectation–maximization Gaussian mixture model, and fast search/density peaks algorithms [27]. The last algorithm was the only one that could maintain large clusters with irregular shapes (Figs S4 and S5B). Two clusters achieved > 0.8 cluster purity for high‐grade prostate cancer, suggesting that these particle populations are found at higher levels within high‐grade prostate cancer patients (Fig. S5C). Although these results appear promising for discrimination, the non‐reproducible nature of viSNE plots requires all data to be analysed simultaneously. Furthermore, as viSNE (through cyt software) can only handle up to 100 000 events, more than 99.99% of particles in our model cohort would be excluded from the analysis. More practical and inclusive analysis methods are necessary for larger clinical studies capable of detecting rare EV particle populations.

### 3.3 Gating microflow cytometry data using gradient‐boosting, decision tree ensemble‐based algorithms provided superior predictive performance

**Fig. 4:**
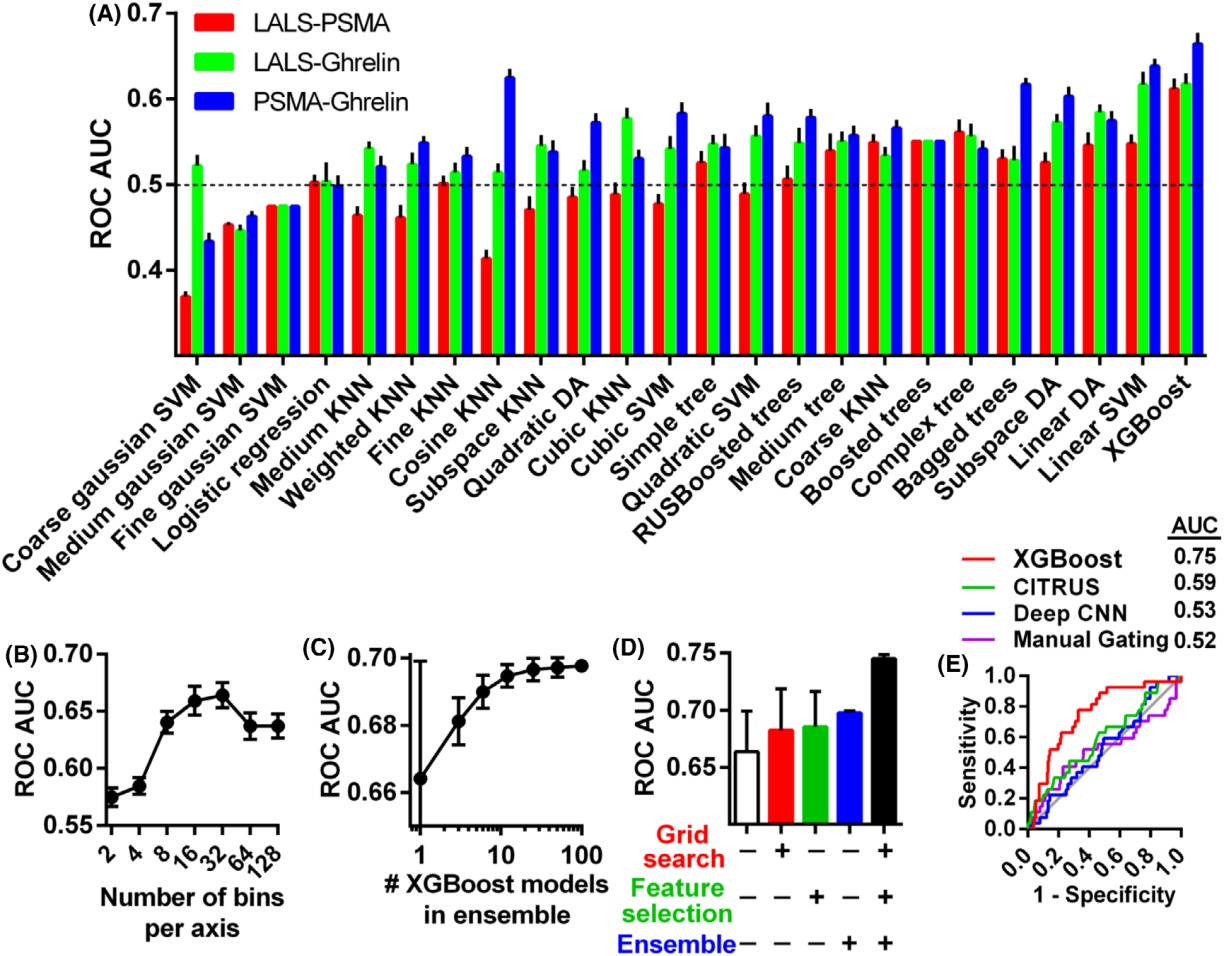
Optimizing extracellular vesicle (EV) machine learning platform (EVMAP) predictive models to predict high‐grade prostate cancer. (A) Comparison of a panel of machine learning algorithms, validated with five‐fold cross‐validation, on the multiple EV microflow cytometry (μFCM) data sets. (B) Optimizing the number of bins per optical parameter. (C) Optimizing the number of XGBoost models in an ensemble. (D) The effect of grid searching XGBoost parameters, feature selection, and ensembling on model performance. (E) Receiver operator characteristic curves for manual gating, Deep CNN, CITRUS and our purpose‐built binning‐XGBoost algorithm to generate the disease prediction score. Plotted values represent mean ± SEM with at least 10 repeats of cross‐validation.

Particle concentrations from ROIs were used as training data for 24 different machine learning algorithms to optimize the prediction of high‐grade prostate cancer from μFCM data. Machine learning algorithms, summarized in Table S4, were validated using five‐fold cross‐validation as described in the Methods. For LALS‐PSMA, LALS‐ghrelin and PSMA‐ghrelin data sets, an algorithm called XGBoost [28] provided the highest AUCs at 0.61, 0.62 and 0.66 respectively (Fig. 4A). We then optimized the data structuring and parameters to improve the predictive performance. As expected for a decision tree‐based model, monotonic transformations of the μFCM data did not improve XGBoost model performance (Fig. S6). The XGBoost gain map, which displays the essential ROIs for XGBoost model accuracy, illustrated that many different particle populations contribute to the overall XGBoost model (Fig. S7A). The ROIs with relatively high gain mostly overlapped with regions on the AUC map that were significantly higher and lower than 0.5, suggesting that particle populations that significantly increased or decreased in high‐grade prostate cancer patients were necessary for the model (Fig. S7B).

We then evaluated the impact of bin size on the model performance. Changing the μFCM binning strategy to above or below 32 bins per parameter caused AUCs to decrease, suggesting that this level of resolution was optimal (Fig. 4B). We examined how the number of models used in the final ensemble affected classification performance and generally found that averaging the predictions from more XGBoost models increased the final AUC (Fig. 4C). Compared to single XGBoost models, an ensemble of 100 models provided a 5% improvement in AUC and reduced model performance variability by 95%. Increasing the number of models beyond 100 continued to increase performance, although the incremental increase in accuracy comes with a high cost in processing and memory requirements with diminishing increases in AUC. In addition, we found that applying a grid searching approach to XGBoost's parameters increased the AUC by 3% (Fig. 4D). We achieved another 3% increase in AUC by employing recursive feature elimination (Fig. 4D). When we used ensembling, grid searching and recursive feature selection together, we found that model AUC increased by 12% (*P* < 0.05), suggesting an additive interaction between model optimization techniques.

In addition, we compared our optimized XGBoost model to the CITRUS algorithm and the Deep Convolutional Neural Network (Deep CNN) method, two leading approaches to predicting clinical outcomes from flow cytometry data. CITRUS uses hierarchical clustering on multi‐dimensional flow cytometry data to identify clusters of events that significantly differ between patient groups [29, 33]. Deep CNN uses a three‐layer neural network on cytometry data and was previously used to accurately identify biomarkers for latent cytomegalovirus infection and other diseases [34]. Compared to the fully optimized XGBoost model, which provided an AUC of 0.75 for the prediction of high‐grade prostate cancer, CITRUS, Deep CNN or manual gating analysis of the PSMA‐ghrelin data set provided significantly lower AUCs of 0.59, 0.53 and 0.52 respectively (*P* < 0.05) (Fig. 4E). Our model also outperformed PSA, which was the only clinical feature that differed significantly between high‐grade and low‐grade prostate cancer patients (*P* = 0.0015) (Table S5).

**Fig. 5:**
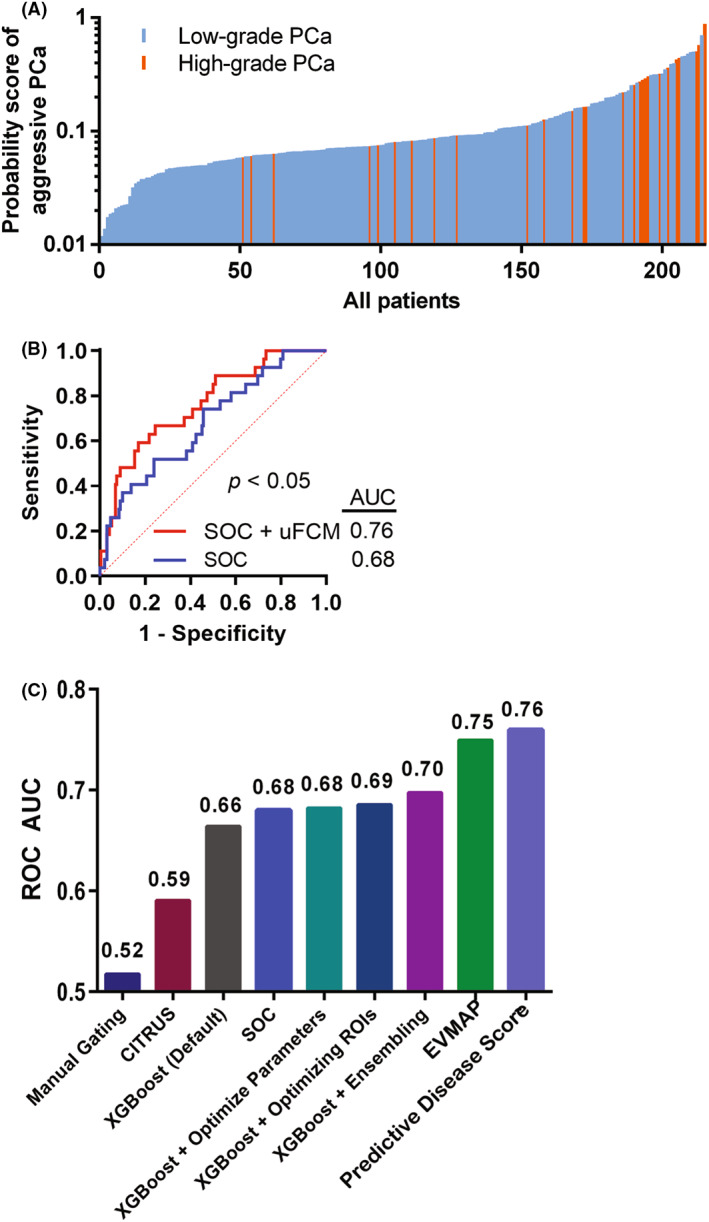
Machine learning analysis of patient standard‐of‐care (SOC) clinical features and microflow cytometry (μFCM) data to predict high‐grade prostate cancer. (A) Waterfall plot of predictions of high‐grade prostate cancer from a logistic regression model using μFCM‐based XGBoost (EVMAP) predictions and patient SOC including PSA, age, race, digital rectal examination (DRE), previous negative biopsy and prostate cancer family history. (B) Receiver operator characteristic (ROC) curves of logistic regression models of SOC with or without EVMAP predictions. (C) Comparison of different model ROC area under the curve values for the prediction of high‐grade prostate cancer.

We created logistic regression models using six standard‐of‐care (SOC) patient‐centric clinical features with or without our μFCM‐based model predictions to compare our optimized model with the SOC features to predict high‐grade prostate cancer. A waterfall plot [35] of patient predictions from the SOC plus μFCM model provided 89% sensitivity and 49% specificity when using a cut‐off probability of 7.332% (Fig. 5A and Table S5). Combined SOC plus μFCM predictions resulted in an AUC of 0.76, which was more significant than the 0.68 AUC from SOC alone for this data set (*P* < 0.05) (Fig. 5B,C). The EVMAP diagnostic platform with custom‐built XGBoost machine learning analysis shows great promise for clinical utility as a predictive test for high‐grade prostate cancer.

### 3.4 EVMAP prostate test would decrease unnecessary biopsies, particularly in men with enlarged prostates

Based on current clinical practice in Alberta, men primarily receive prostate biopsies due to high PSA levels and/or abnormal DREs [36]. However, about 50% of men aged 51–60 and up to 90% of men older than 80 develop a benign condition where their prostate is enlarged [37]. Men with enlarged prostates in the model 215 patient cohort were significantly less likely to have prostate cancer. The fraction of patients with abnormal DRE was similar between men with normal and enlarged prostates (Fig. S8A) while PSA levels were significantly higher in men with enlarged prostates (*P* < 0.05) (Fig. S8B). Therefore, more men with enlarged prostates underwent unnecessary biopsies compared to men with normal‐sized prostates. Normalizing PSA levels using PSA density (PSA divided by prostate volume) may not be ideal since PSA density was significantly lower in men with enlarged prostates (Fig. S8C). For men with enlarged prostates, their disease scores were significantly different between low‐grade and high‐grade prostate cancer patients (*P* < 0.0005) (Fig. S8D). Using the previously defined probability cutoff threshold in Table S5, 100% of patients with high‐grade prostate cancer and 49% of patients with low‐grade prostate cancer would be recommended for biopsy. This eliminates approximately half of unnecessary biopsies while still maintaining 100% sensitivity for detecting high‐grade prostate cancer (Fig. S8E).

## 4 Discussion

High‐resolution microflow cytometry of EVs is a relatively recent innovation with broad potential for clinical utility. While continual hardware improvements have allowed the detection and characterization of individual EVs, lipoproteins and protein aggregates as small as 50 nm [38], improvements in the analysis of complex μFCM data have been lacking. Future improvements in instrument resolution using light scatter and fluorescent sensitivity will have to balance sample complexity with the ratio of true signal to noise. This will necessitate a significant sample dilution and decrease in sheath pressure as seen with some instruments available today. Balancing sheath pressure with sample flow rate may in turn create technical challenges for flow stability and sample throughput. Ensuring that after all of the technical challenges, enough true sample signal is interrogated to provide an accurate representation of the population will be critical for data quality. To this end, different perspectives for the analysis of extracellular vesicles by flow cytometry should be investigated. Our results highlight the importance of thorough μFCM data analysis using the whole EV population to capture the wealth of information missed using conventional gating approaches.

While the fixed manual gating strategy used in this study allowed rapid, consistent and straight‐forward analysis, it may not have provided the optimal results for each patient's FCS files. Dynamic gating strategies for each FCS file based on unstained controls may have provided better results than those shown. However, it is highly unlikely that manual gating would be equal or superior to EVMAP in this application given the drastic difference in clinical performance between the two analysis strategies. The relative performance of manual gating compared to EVMAP will be dependent on the specific assay and application.

By using a rapid binning strategy to divide μFCM‐quantified EV particle data into discrete populations, EVMAP allowed all data from the patient sample to be used instead of a subjectively defined area. A patient flow cytometry standard (FCS) file with 5 000 000 events can be read, log‐transformed, events assigned to ROIs and particle concentration determined for each ROI in under 14 s using a single thread of an i7‐6700K CPU. In contrast, we found that clustering algorithms such as CITRUS [29, 33], SWIFT [33, 39] and SPADE [33, 40] required several hours or days to cluster the 215 patient data set. Unlike SPADE and Deep CNN, our binning method does not remove any data from analysis via downsampling, allowing detection of ultra‐rare particle populations. New data can also have events easily assigned to ROIs using binning, allowing processing and predictions for future patients with previously analysed data sets. The limitations of our binning approach (and of many other clustering algorithms) include the requirement for minimal changes in event signal intensities and an exponential increase in ROI number as additional biomarkers are included. When clustering events using binning, the total number of ROIs is equal to the number of bins per marker to the power of the number of biomarkers. If more than three biomarkers are analysed simultaneously, multi‐dimensional clustering algorithms are recommended to minimize the number of features used for machine learning. Future work comparing a wider variety of clustering algorithms is warranted.

Upon extracting feature data from μFCM data, various machine learning algorithms can be used to predict patient clinical status. The CITRUS algorithm uses shrunken centroid and lasso‐regularized logistic regression models. While both models are computationally efficient, they are both relatively simplistic without any bagging or boosting mechanisms for increased accuracy. The FloReMi algorithm used a random survival forest model to obtain the highest accuracy for predicting survival times in the FlowCap IV challenge [41]. This model, and the original random forest, bags weaker models together, although it still lacks boosting. The XGBoost algorithm, which performs boosting by iteratively creating larger tree ensemble models with improved accuracy, provides state‐of‐the‐art accuracy on many structured data sets due to its regularization to minimize overfitting and computational optimizations to allow rapid identification of near‐ideal hyperparameters [27, 28].

Significant variability and error are associated with assigning tumour grade groups to prostate biopsies which causes label errors and limits predictive model accuracies. A previous study of concordance between biopsy and surgical prostate cancer grade group from radical prostatectomies illustrates that biopsy grade group has an AUC of 0.80 for predicting ground truth surgical grade group 3 and greater prostate cancer [42]. This ~ 20% mismatch may be due many reasons including biopsy sampling error (i.e. needles missing most relevant tumour regions) as well as inter‐pathologist disagreement. The 0.75 AUC of EVMAP is close to the theoretical maximum AUC of approximately 0.80, demonstrating the relatively high performance of this test.

## 5 Conclusions

The findings of this study show that EVMAP can identify clinically relevant EVs in μFCM data and create state‐of‐the‐art disease prediction models to generate the patient's risk of disease. EVMAP technology will expand EV research and promote the translation of basic research to develop predictive tests for diseases with clinically unmet needs beyond prostate cancers, including other cancers, like bladder cancer, plus cardiac disease, infectious diseases and neurodegenerative diseases.

## Conflict of interest

RJP, DP, CV and JDL are employees and shareholders in Nanostics Inc. DLS has no conflicts of interest to disclose.

## Author contributions

RJP was involved in data curation, formal analysis, investigation, methodology, software, validation, visualization, writing—original draft, and writing—review and editing. DP was involved in data curation, formal analysis, investigation, methodology, validation, visualization, writing—original draft, writing—review and editing. DLS was involved in investigation. CV was involved in data curation, methodology, formal analysis and writing‐original draft. JDL was involved in conceptualization, formal analysis, funding acquisition, resources, project administration, supervision writing—original draft, writing—review and editing.

## Supporting information


**Fig. S1.** Instrument cleaning and assay controls, Levey–Jennings plot.
**Fig. S2.** Fluorescence calibration.
**Fig. S3.** Instrument stability. Instrument stability was assessed by plotting a Levey–Jennings assessment of the daily monitoring beads (Apogee product 1493).
**Fig. S4.** viSNE clustering of μFCM data.
**Fig. S5.** viSNE analysis of μFCM data.
**Fig. S6.** XGBoost model performance predicting high‐grade prostate cancer from μFCM data not affected by monotonic data transformations.
**Fig. S7.** XGBoost gain map and AUC map overlay.
**Fig. S8.** Clinical features and μFCM data to predict high‐grade prostate cancer.
**Table S1.** Microflow cytometry settings.
**Table S2.** MIFlowCyt‐EV/MISEV compliant items for the standardized reporting of extracellular vesicle flow cytometry experiments [23].
**Table S3.** Cohort statistics.
**Table S4.** List of predictive models for grade group ≥3 prostate cancer.
**Table S5.** Patient characteristics and disease prediction scores.

## Data Availability

This study includes FCS file data deposited in external repositories. The supporting data are contained in the manuscript as Supporting Information.
