# Supplementary material for: Building predictive disease models using extracellular vesicle microscale flow cytometry and machine learning
Source: Mol Oncol. 2022 Dec 29;17(3):407–21. doi: 10.1002/1878-0261.13362 (PMC9980304; doi:10.1002/1878-0261.13362)
Supplement: Supplementary file 1 — Fig. S1. Instrument cleaning and assay controls, Levey–Jennings plot. Fig. S2. Fluorescence calibration. Fig. S3. Instrument stability. Instrument stability was assessed by plotting a Levey–Jennings assessment of the daily monitoring beads (Apogee product 1493). Fig. S4. viSNE clustering of μFCM data. Fig. S5. viSNE analysis of μFCM data. Fig. S6. XGBoost model performance predicting high‐grade prostate cancer from μFCM data not affected by monotonic data transformations. Fig. S7. XGBoost gain map and AUC map overlay. Fig. S8. Clinical features and μFCM data to predict high‐grade prostate cancer. Table S1. Microflow cytometry settings. Table S2. MIFlowCyt‐EV/MISEV compliant items for the standardized reporting of extracellular vesicle flow cytometry experiments [23]. Table S3. Cohort statistics. Table S4. List of predictive models for grade group ≥3 prostate cancer. Table S5. Patient characteristics and disease prediction scores. [file MOL2-17-407-s001.docx]

Supplementary Material for

**Building predictive disease models using extracellular vesicle microscale flow cytometry and machine learning.**

Robert J. Paproski^1,2,†^, Desmond Pink^1,2,†^, Deborah L. Sosnowski^1^, Catalina Vasquez^1,2^, John D. Lewis^1,2^*****

†These authors contributed equally to this work as first authors.

^1^Department of Oncology, University of Alberta, Edmonton, Alberta, Canada, T6G 2E1.

^2^Nanostics Inc., 4550 10230 Jasper Avenue, Edmonton, Alberta, Canada, T5J 4P6.

**Keywords:** Diagnostic test, Cancer prediction, Extracellular vesicles, Machine learning, Microflow cytometry, Prostate cancer

**Abbreviations:**

APCaRI, Alberta Prostate Cancer Research Initiative

Deep CNN, Deep Convolutional Neural Network

DRE, digital rectal exam

EV, extracellular vesicle

EVMAP, extracellular vesicle machine learning analysis platform

FCS, flow cytometry standard

FL, fluorescence

LALS, large angle light scatter

MESF, molecules of equivalent soluble fluorophore

MFI, median fluorescent intensity

PCa, prostate cancer

PSA, prostate-specific antigen

PSMA, Prostate-Specific Membrane Antigen

ROC AUC, receiver operator characteristic area under the curve

RI, refractive index

ROI, regions of interest

SOC, standard-of-care

µFCM, microflow cytometry

XGBoost, Extreme Gradient Boosting


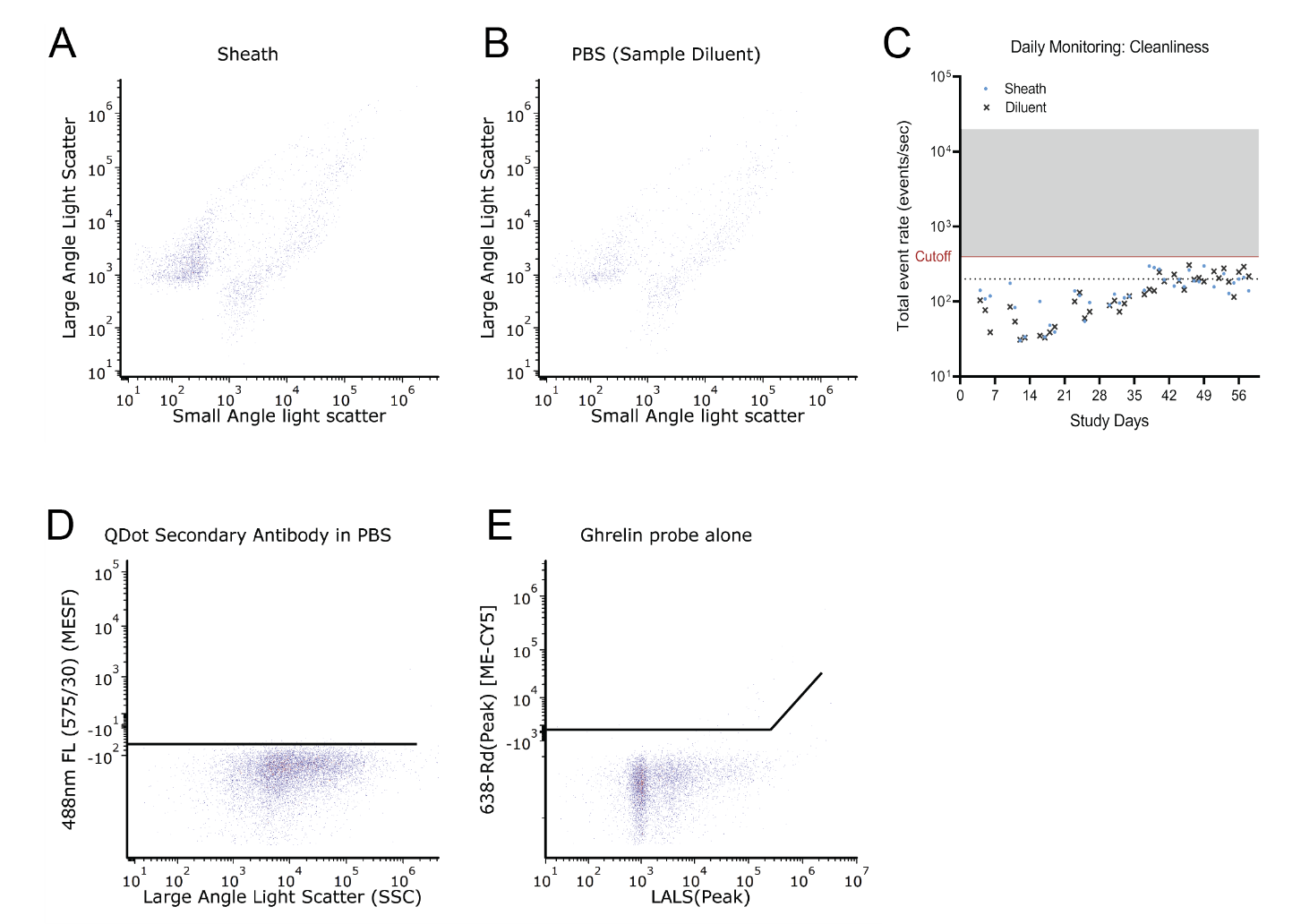


**Fig. S1.**

Instrument cleaning and Assay Controls, Levey-Jennings plot. A,B,C) Assay controls (secondary) antibody or peptide probe alone in PBS were assessed following the same conditions as the nominal without sample being added (D, E).

**
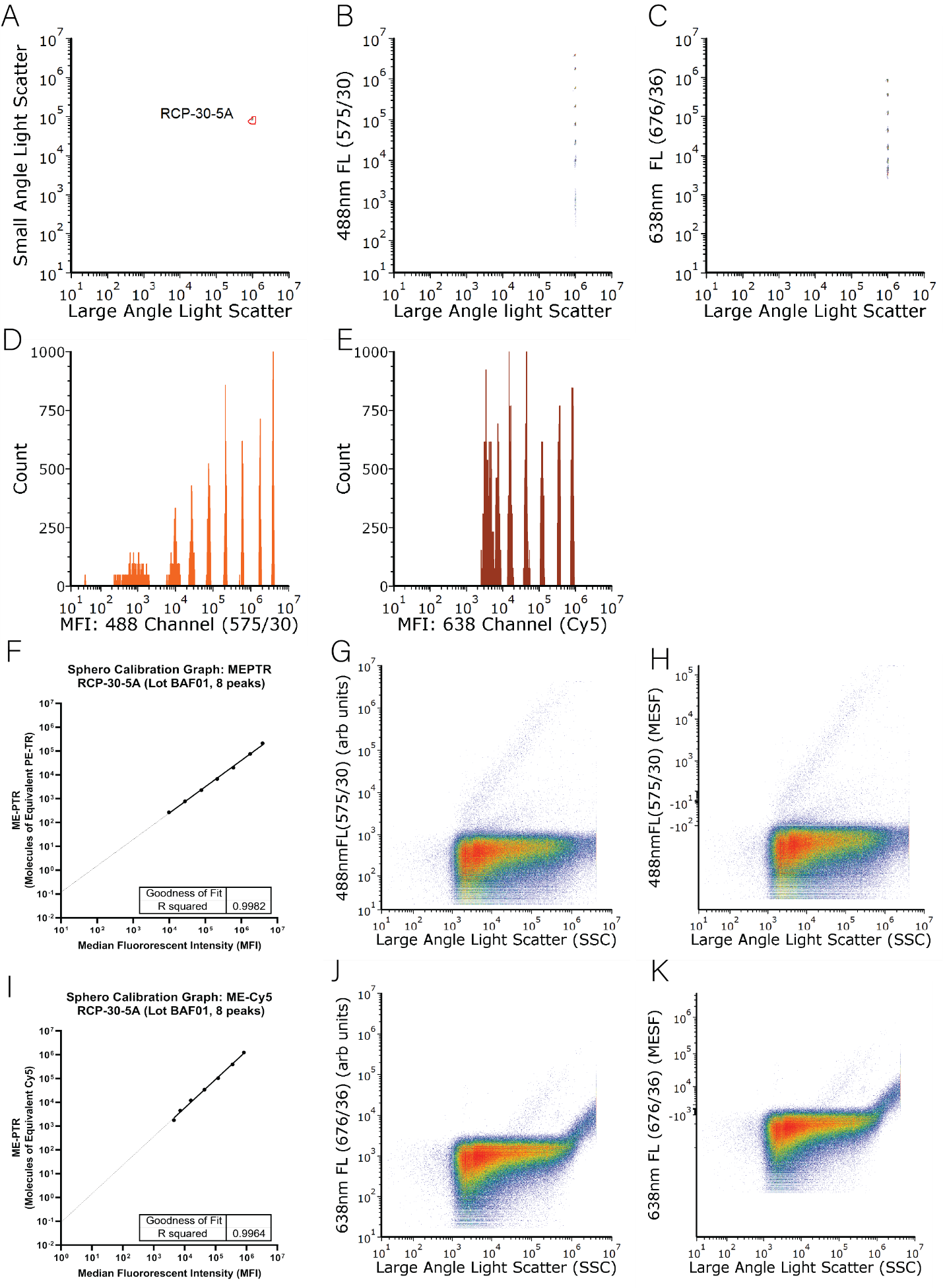
**

**Fig S2.**

Fluorescence sensitivity was calibrated using Spherotech reference beads (RCP-30-5A, LotBAF01) and the related data presented in Figure S1. The multi bead population was gated (red circle, A) on a graph of Large Angle Light Scatter (SSC) versus Small Angle Light Scatter (FSC). The gated particles were then graphed first by the 488 excited fluorescence (B) or 638 excited fluorescence (C) by SSC to illustrate distinct bead populations. The same data were plotted on histograms (D, E). The log transformations of the specified molecules of equivalent soluble fluorophore (MESF) values versus log transformed of the associated median fluorescent intensities (MFI) for MEPTR (F) and ME-Cy5 (I). Both transformations had goodness of fit values >0.99. The slope of the line and intercept values are used to transform FL intensity data to MESF equivalents using the illustrated formula where *Int*_MESF_= calibrated intensities, a is the slope of the regression line, b is the intersect and Int_measured_ are the measured fluorescent intensities.

$${Int}_{MESF}= {10}^{a\cdot{log}_{10} \left( {Int}_{measured} \right)+b}$$

A representative sample is provided to illustrate the uncalibrated (G,J) and calibrated data transformations (H,K).


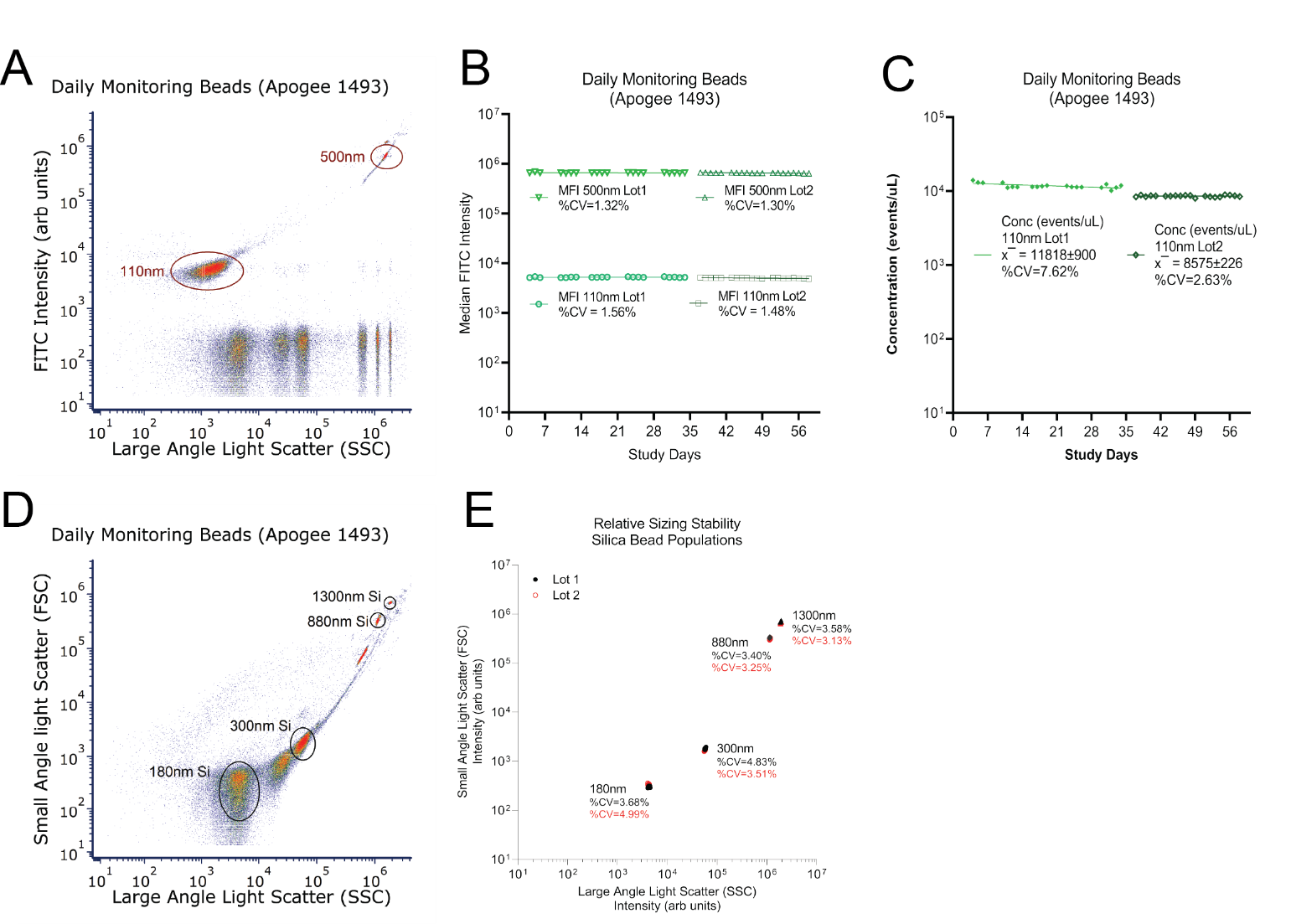


**Fig. S3.** Instrument stability was assessed by plotting a Levey-Jennings assessment of the daily monitoring beads (Apogee product 1493). The 488nm FITC versus SSC plot of the monitoring beads (A) shows the two polystyrene populations (110nm and 500nm, red gates) as well as the six silica bead populations (180nm, 240nm, 300nm, 590nm, 880nm, and 1300nm). Relative fluorescence stability was gauged by plotting the MFI of the 110nm and 500nm polystyrene beads which contain 488nm excited dye detected in the FITC channel. The data from two separate lots of bead preparations are shown (n=20/lot,). Median FL intensities show little variability with %CV for each data set <2% (B). While bead concentrations are slightly different for each lot (expected), the reproducibility (%CV) of counts was 7.62% for Lot 1 and 2.63% for Lot2 (C). The sizing of the detected events is described relative to the size of the different monitoring beads in the Apogee 1493 mixture. To assess the consistency of this relative measure, the median intensity of the bead populations indicated (D) for both FSC and SSC were compared for the period of the study (~2 months). The variability (%CV) for bead populations is indicated (E) and is consistently below 5%. The data presented in this paper indicate that the biological events detected ranged in size, approximating the range between 110nm polystyrene to ~500nm polystyrene.


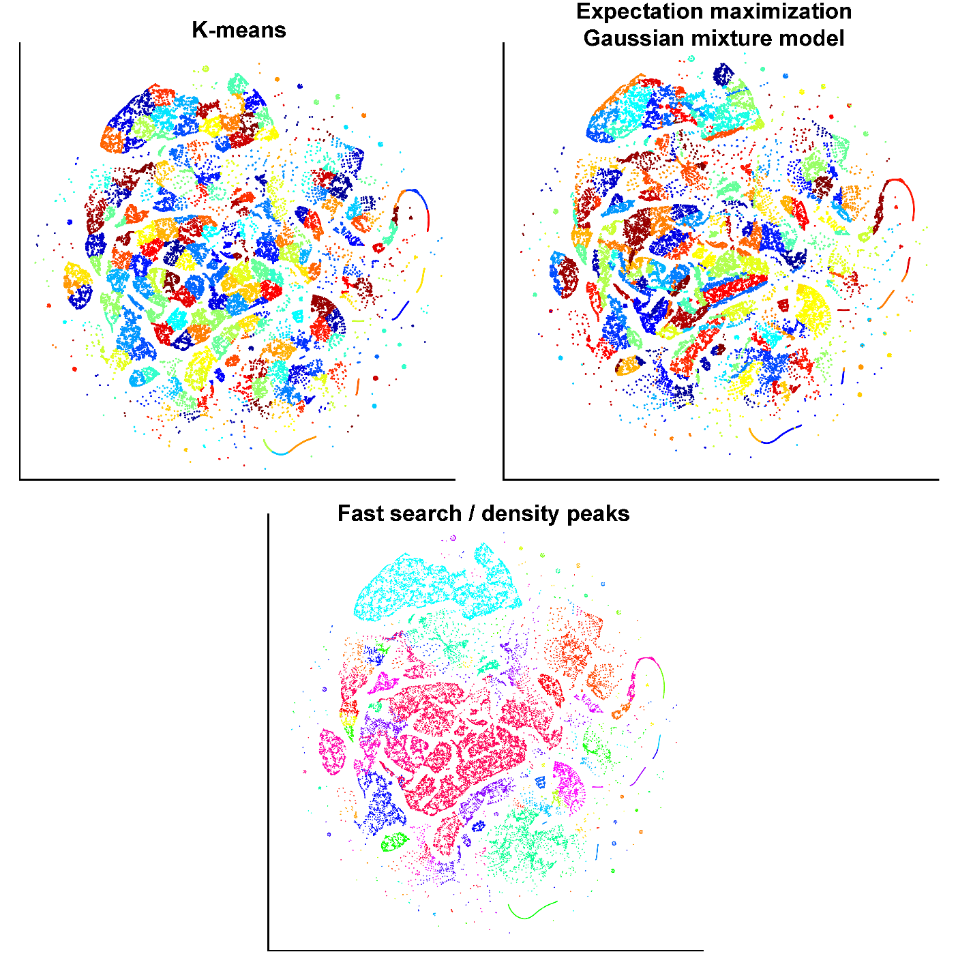


**A**

**B**

**C**

Fig. S4. viSNE clustering of µFCM data. An equal number of particles (30,000) from high-grade and low-grade prostate cancer patients were transformed with viSNE and clustered using A) K-means, B) Expectation maximization Gaussian mixture model, and C) fast search/density peaks methods.


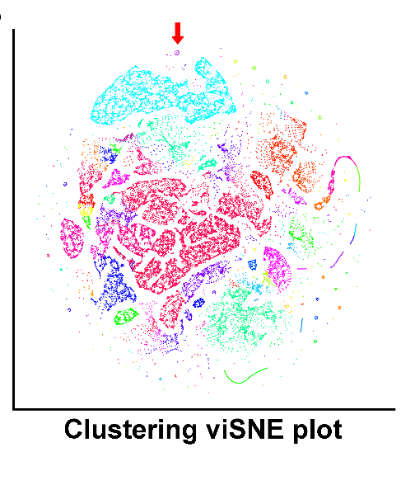

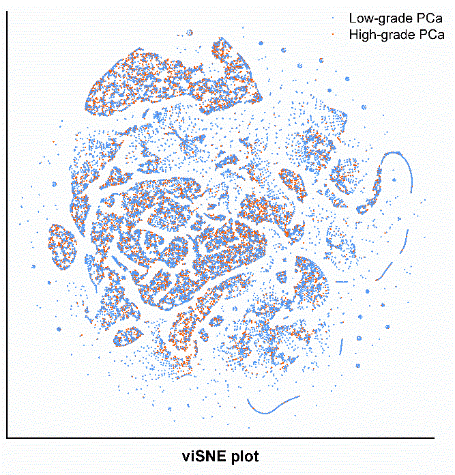


**B**

**A**





**C**

Fig. S5. viSNE analysis of µFCM data. A) An equal number of particles (30,000) from high-grade and low-grade prostate cancer patients were analyzed with viSNE. B) Particles were clustered using the fast search/density peaks algorithm. C) viSNE cluster purity for high-grade prostate cancer particles. Some clusters show enrichment for particles derived from high-grade prostate cancer patients (red arrow).





Fig. S6. XGBoost model performance predicting high-grade prostate cancer from µFCM data not affected by monotonic data transformations.

**
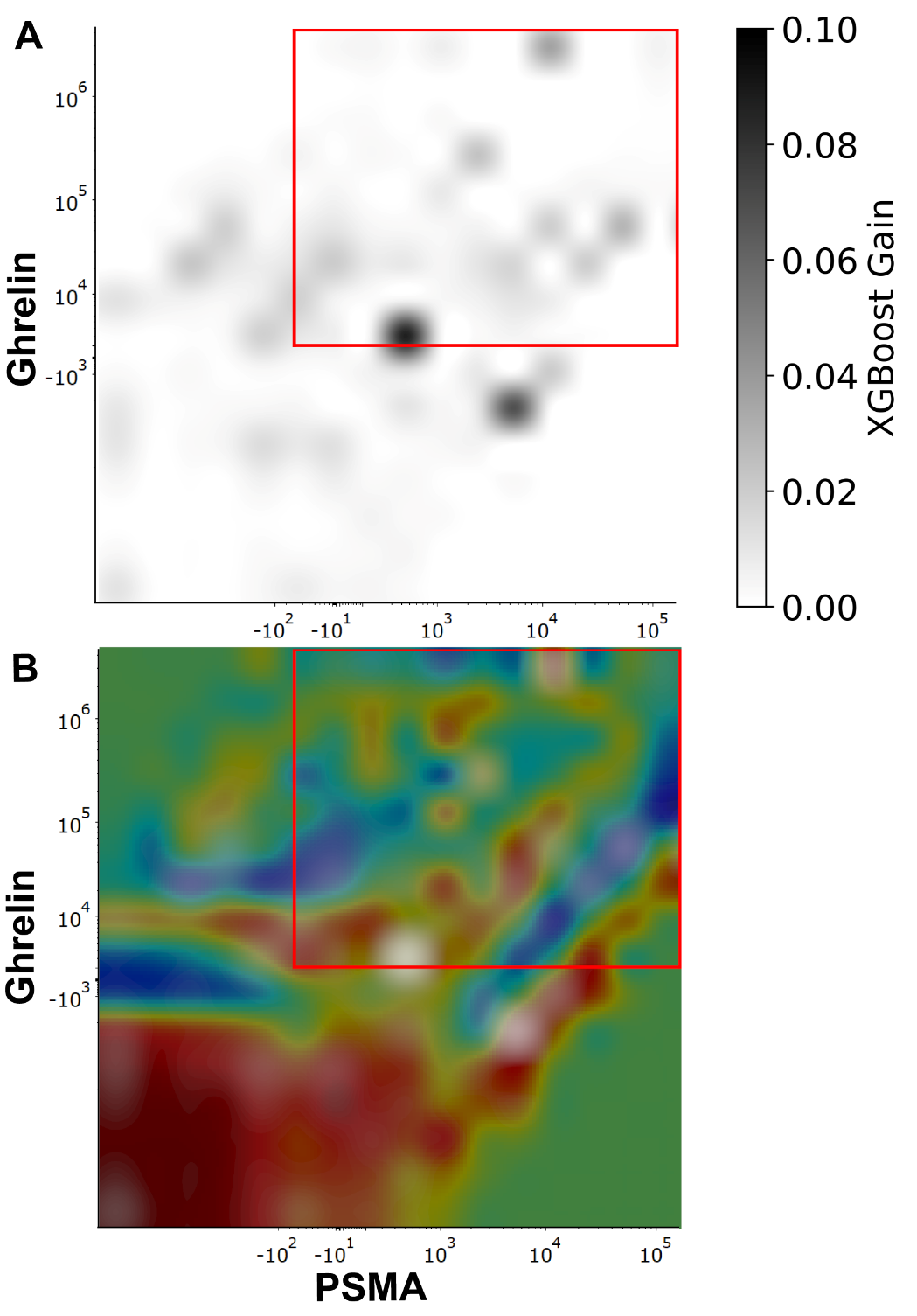
**

**Fig. S7.** XGBoost gain map and AUC map overlay. A) XGBoost gain map, which displays the improvement in accuracy by each ROI for the XGBoost model, illustrated that many different particle populations contribute to the overall XGBoost model. B) The ROIs with relatively high gain mostly overlapped with regions on the AUC map that were significantly higher and lower than 0.5, suggesting that particle populations that significantly increased or decreased in high-grade prostate cancer patients were necessary for the model.


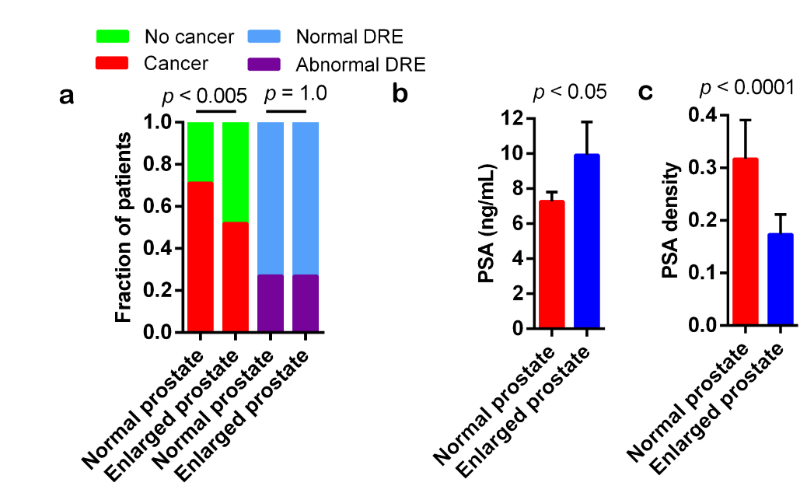


**C**

**B**

**A**

**

**

**E**

**

**

**D**

**Fig. S8.** Clinical features and µFCM data to predict high-grade prostate cancer. A) Fraction of patients with or without enlarged prostates (>40cc) with a cancer diagnosis and abnormal DRE. B) PSA and C) PSA density in men with and without enlarged prostates. Plotted values are mean ± SEM. D) Predictions of high-grade prostate cancer in men with enlarged prostates using EVMAP + SOC logistic regression model. E) Recommendation of whether men with enlarged prostates should receive a biopsy with the EVMAP+SOC score.

**Table S1.** Microflow cytometry settings.

| **Platform** | Apogee A50 MP | S/N 0027 |  |  |
| --- | --- | --- | --- | --- |
| **Parameter** | **Setting** |  | Antibody volume | 3 to 5 µL each |
| Sample Flow Rate | 3.01 µL/min |  |  |  |
| Pressure | 150 units |  |  |  |
| Acquisition time | 60 sec |  |  |  |
| Sample Dilution | 100x with PBS |  |  |  |
| Volume/well | 250 µL |  |  |  |
| Sample volume | 10 µL |  |  |  |
| Diluent volume | 990- Ab vol µL |  |  |  |
| Event Trigger | LALs unless stated otherwise | |  |  |
|  |  |  |  |  |
| **Channel** | **Laser Power (mW)** | **PMT** | **Gain** | **Threshold** |
| 405nm | 75 |  |  |  |
| 488nm | 50 |  |  |  |
| 561nm | N/A |  |  |  |
| 638nm | 75 |  |  |  |
| 405-SALS |  | 342 | 1.0 | 20 |
| 405-LALS |  | 350 | 1.0 | 48 |
| 405-Red |  | 350 | 1.0 |  |
| 405-Green |  | 500 | 1.0 |  |
| 488-Green |  | 450 | 1.0 |  |
| 488-Orange |  | 550 | 1.0 |  |
| 488-Red |  | 580 | 1.0 |  |
| 561-Orange |  | NA | 1.0 |  |
| 561-Red |  | NA | 1.0 |  |
| 638-Red |  | 550 | 1.0 |  |
| 638-Far Red |  | NA | 1.0 |  |
|  |  |  |  |  |
| **Beads** | **Product number** | **Lot number** | **Expiration** |  |
| Monitoring | Apogee 1493 | CAL0093  CAL0095 | 09/08 2022  13/02/2023 |  |

**Table S2.** MIFlowCyt-EV/ MISEV compliant items for the standardized reporting of extracellular vesicle flow cytometry experiments [39].

| **Requirement** | **Requested Information** |
| --- | --- |
| 1.1. Purpose | To assess the use of liquid biopsy (platelet depleted plasma) as a source of extracellular vesicles for detection of different prostate cancer biomarkers. |
| 1.2. Keywords | Prostate cancer, plasma, biomarker, extracellular vesicle |
| 1.3. Experiment variables | Patient plasma, risk of cancer. Specific details in Methods. |
| 1.4. Organization name and address | John D. Lewis Lab, University of Alberta, Dept of Experimental Oncology, 5142 Katz Group Centre Edmonton, Alberta, Canada T6G 2E1 |
| 1.5. Primary contact name and email address | John D. Lewis, jdlewis@ualberta.ca |
| 1.6. Date or time period of experiment | 2017 - 2020 |
| 1.7. Conclusions | Our data demonstrate that extracellular vesicles, in this primary study may be useful for the detection of prostate cancer and in conjunction with machine learning algorithms may be predictive or high-grade cancer risk. |
| 1.8. Quality control measures | 1493 Apogee Bead Mix, daily instrument monitoring. |
| 2.1.1.1. (2.1.2.1., 2.1.3.1.) Sample description | Platelet-free plasma from patients at risk for prostate cancer. |
| 2.1.1.2. Biological sample source description | Pre-biopsy plasma samples from 215 men suspected of prostate cancer were acquired from the Alberta Prostate Cancer Research Initiative (APCaRI) biorepository(35). The clinical study was approved by the Health Research Ethics Board of Alberta under the APCaRI-01 protocol (HREBA-CC-18-0513). |
| 2.1.1.3. Biological sample source organism description | Human, EDTA plasma |
| 2.1.2.2. Environmental sample location | NA |
| 2.3. Sample treatment description | Human plasma samples   1. Frozen plasma samples were thawed, centrifuged at 16,000xg for 30 minutes to remove large debris and platelet particles, and incubated with five µg/mL mouse anti-PSMA (J591) antibody and a 1:50 dilution of secondary Qdot565-conjugated donkey anti-mouse IgG antibody. 2. Samples were also incubated with 0.133 mM Cy5.5-ghrelin probe containing the first 18 amino acids of ghrelin. 3. Thirty minutes after probe incubation, samples were diluted 100-fold in double-filtered (0.1 µm) phosphate-buffered saline and analyzed with the Apogee A50 microflow cytometer using a flow rate of 3.01 µL/minute. Samples were run for up to 2 minutes or until 5,000,000 events were recorded, whichever came first. 4. Plasma from each patient was run in triplicate. Conventional manual gating analysis of µFCM data was performed using Histogram version 255.0.0.80 software (Apogee Flow Systems). |
| 2.4. Fluorescence reagent(s) description | 1. Secondary Qdot565-conjugated donkey anti-mouse IgG antibody. 2. 0.133 mM Cy5.5-ghrelin probe |
| 3.1. Instrument manufacturers, model | 1. Apogee A50 MicroPlus Microflow cytometer (S/N 0027): Apogee Flow Systems |
| 3.3. Instrument configuration and settings | See Tables |
| 4.1. List-mode data files | 2) The repository identifier:  http://flowrepository.org/id/FR-FCM-xxxx (copy and paste the code). This link will be made publicly accessible after the paper is published. |
| 4.2. Compensation description | No compensation |
| 4.3. Data transformation details | NA |
| 4.4.1. Gate description | Defined by unstained controls. |
| 4.4.2. Gate statistics | Data provided as concentration (events/uL) |
| 4.4.3. Gate boundaries | Defined by unstained controls as well as autogating defined by ML algorithms |

**Table S3.** Cohort statistics.

| Total patients | 215 | |
| --- | --- | --- |
| Age, years |  | |
| Median, (IQR) | 62 (57 - 68) | |
| Unknown, n | 0 | |
| PSA, ng/mL |  | |
| Median, (IQR) | 6.2 (4.7 - 8.7) | |
| Unknown, n | 1 | |
| Race, n (%) |  | |
| Non-Black | 179 (97.8%) | |
| Black | 4 (2.2%) | |
| Unknown | 32 | |
| Digital rectal exam findings, n (%) |  | |
| Normal | 116 (71.6%) | |
| Abnormal | 46 (28.4%) | |
| Unknown | 53 | |
| Prior negative biopsy, n (%) |  | |
| No | 180 (90.5%) | |
| Yes | 19 (9.5%) | |
| Unknown | 16 | |
| Family history prostate cancer, n (%) |  | |
| No | 111 (68.9%) | |
| Yes | 50 (31.1%) | |
| Unknown | 54 | |
| Biopsy results, n (%) |  | |
| Negative Biopsy | 82 (38.1%) | |
| Grade Group 1 | 59 (27.4%) | |
| Grade Group 2 | 47 (21.9%) | |
| Grade Group 3 | 16 (7.4%) | |
| Grade Group 4 | 5 (2.3%) | |
| Grade Group 5 | 6 (2.8%) | |
| Unknown | 0 | |
| Tumor stage, n (%) |  | |
| Stage 1 | 94 (70.7%) | |
| Stage 2 | 35 (26.3%) | |
| Stage 3 | 3 (2.3%) | |
| Stage 4 | 1 (0.8%) | |
| Unknown | 82 | |
| High grade prostatic intraepithelial neoplasia, n (%) |  | |
| No | 58 (37.4%) | |
| Yes | 97 (62.6%) | |
| Unknown | 60 | |
| Perineural invasion, n (%) |  | |
| No | 99 (68.3%) | |
| Yes | 46 (31.7%) | |
| Unknown | 70 | |
| PSA: Prostate specific antigen | |  |
| Percentages based on available data and may not sum to 100% due to rounding | |  |

| **Table S4.** List of predictive models for grade group ≥3 prostate cancer. | | | |  |
| --- | --- | --- | --- | --- |
| **Model ID** | **Name** | **Figures** | **Algorithm** | **Hyperparameters + model optimizations** |
| 1 | Coarse gaussian SVM | 4A | Support vector machine | Default Matlab's Classification Learner parameters^1^ |
| 2 | Medium gaussian SVM | 4A | Support vector machine | Default Matlab's Classification Learner parameters^1^ |
| 3 | Fine gaussian SVM | 4A | Support vector machine | Default Matlab's Classification Learner parameters^1^ |
| 4 | Logistic regression | 4A | Logistic regression | Default Matlab's Classification Learner parameters^1^ |
| 5 | Medium KNN | 4A | K-nearest neighbors | Default Matlab's Classification Learner parameters^1^ |
| 6 | Weighted KNN | 4A | K-nearest neighbors | Default Matlab's Classification Learner parameters^1^ |
| 7 | Fine KNN | 4A | K-nearest neighbors | Default Matlab's Classification Learner parameters^1^ |
| 8 | Cosine KNN | 4A | K-nearest neighbors | Default Matlab's Classification Learner parameters^1^ |
| 9 | Subspace KNN | 4A | K-nearest neighbors | Default Matlab's Classification Learner parameters^1^ |
| 10 | Quadratic DA | 4A | Discriminant analysis | Default Matlab's Classification Learner parameters^1^ |
| 11 | Cubic KNN | 4A | K-nearest neighbors | Default Matlab's Classification Learner parameters^1^ |
| 12 | Cubic SVM | 4A | Support vector machine | Default Matlab's Classification Learner parameters^1^ |
| 13 | Simple tree | 4A | Decision tree | Default Matlab's Classification Learner parameters^1^ |
| 14 | Quadratic SVM | 4A | Support vector machine | Default Matlab's Classification Learner parameters^1^ |
| 15 | RUSBoosted trees | 4A | Random Undersampling Boosting decision trees | Default Matlab's Classification Learner parameters^1^ |
| 16 | Medium tree | 4A | Decision tree | Default Matlab's Classification Learner parameters^1^ |
| 17 | Coarse KNN | 4A | K-nearest neighbors | Default Matlab's Classification Learner parameters^1^ |
| 18 | Boosted trees | 4A | Boosted decision trees | Default Matlab's Classification Learner parameters^1^ |
| 19 | Complex tree | 4A | Decision tree | Default Matlab's Classification Learner parameters^1^ |
| 20 | Bagged trees | 4A | Bagged decision trees | Default Matlab's Classification Learner parameters^1^ |
| 21 | Subspace DA | 4A | Discriminant analysis | Default Matlab's Classification Learner parameters^1^ |
| 22 | Linear DA | 4A | Discriminant analysis | Default Matlab's Classification Learner parameters^1^ |
| 23 | Linear SVM | 4A | Support vector machine | Default Matlab's Classification Learner parameters^1^ |
| 24 | XGBoost (Default) | 4A, 4D (white bar), 5C | XGBoost | Default XGBoost parameters^2^ |
| 25 | XGBoost (Optimizing ROIs) | 4B, 5C | XGBoost | Default XGBoost parameters^2^ with different binning of microflow data |
| 26 | XGBoost + Ensembling | 4C, 4D (blue bar), 5C | XGBoost | *Default XGBoost parameters^2^ with different number of ensembled XGBoost models |
| 27 | XGBoost (Optimize Parameters) | 4D (red bar), 5C | XGBoost | Grid search best XGBoost parameters^3^ |
| 28 | XGBoost | 4D (green bar) | XGBoost | Default XGBoost parameters^2^ with feature selection by recursive feature elimination |
| 29 | XGBoost (EVMAP) | 4D (black bar), 4E, 5C, S3, S4 | XGBoost | Grid search best XGBoost parameters^3^ + feature selection with recursive feature elimination + 100 ensembled XGBoost models |
| 30 | CITRUS | 4E, 5C | CITRUS | Default CITRUS parameters^4^ |
| 31 | Deep CNN | 4E | Deep CNN | Default Deep CNN parameters^5^ |
| 32 | SOC | 5B, 5C | Logistic regression | R, caret package, method=glm, family=binomial (link=logit)^6^ |
| 33 | SOC + μFCM (Predictive Disease Score) | 5A, 5B, 5C, S5D, S5E | Logistic regression | R, caret package, method=glm, family=binomial (link=logit)^6^ |
| SVM: Support vector machine | | | | |
| KNN: K-nearest neighbors | | | |  |
| DA: Discriminant analysis | | | |  |
| XGBoost: eXtreme Gradient Boosting | | | |  |
| CITRUS: Cluster identification, characterization, and regression | | | |  |
| CNN: Convolutional neural network | | | |  |
| SOC: Standard of care | | | |  |
| ^1^ https://www.mathworks.com/help/stats/classificationlearner-app.html | | | | |
| ^2^ Default XGBoost parameters: objective=binary logistic, max_depth=6, eta=0.01, gamma=0, colsample_bytree=1, min_child_weight=1, subsample=1 | | | | |
| ^3^ Grid search XGBoost parameters by AUC: {nrounds = 50, 100, 150, 200, 250, 300, 400; max_depth = 3, 4, 5, 6; eta = 0.01, 0.1} | | | | |
| ^4^ https://rdrr.io/github/nolanlab/citrus/src/R/citrus.R | | | | |
| ^5^ https://github.com/hzc363/DeepLearningCyTOF | | | | |
| ^6^ https://daviddalpiaz.github.io/r4sl/the-caret-package.html | | | | |

| **Table S5.** Patient characteristics and disease prediction scores. | | | | | | | | |  |  |  | |  |  | |  | |  |
| --- | --- | --- | --- | --- | --- | --- | --- | --- | --- | --- | --- | --- | --- | --- | --- | --- | --- | --- |
| **Patient characteristics by prostate cancer grade group** | **Grade Group < 2** | **Grade Group > 3** | **p-value** | **ROC AUC mean (CI)** | **Cutoff (>)** | | | **Sens (CI)** | | | | **Spec % (CI)** | | | **PPV (CI)** | | **NPV % (CI)** | |
| Patients, n | 188 | 27 |  |  |  | | |  | | | |  | | |  | |  | |
| Race, n (% black) | 3 (1.6) | 1 (3.7) | 0.42 | 0.51 (0.39-0.63) | - | | | 3.7 (0.094-19) | | | | 98 (95-100) | | | 25 (0.63-81) | | 88 (82-92) | |
| Family history of PCa, n (%) | 53 (29) | 6 (22) | 0.65 | 0.53 (0.42-0.65) | - | | | 22 (8.6-42) | | | | 71 (64-78) | | | 10 (3.8-21) | | 86 (80-91) | |
| Previous negative biopsy, n (%) | 20 (11) | 1 (3.7) | 0.49 | 0.54 (0.42-0.65) | - | | | 3.7 (0.094-19) | | | | 89 (84-93) | | | 4.8 (0.12-24) | | 86 (81-91) | |
| DRE, n (% abnormal) | 48 (26) | 10 (37) | 0.25 | 0.56 (0.44-0.68) | - | | | 37 (19-58) | | | | 74 (67-80) | | | 17 (8.6-29) | | 89 (83-93) | |
| Age, yr, mean (CI) | 62 (60-63) | 65 (61-68) | 0.2 | 0.58 (0.45-0.70) | 53.95 | | | 89 (71-98) | | | | 14 (9.7-20) | | | 13 (8.5-19) | | 90 (73-98) | |
| PSA, ng/ml, mean (CI) | 7.4 (6.2-8.7) | 16 (3.8-29) | **0.0015** | 0.69 (0.58-0.79) | 5.25 | | | 89 (71-98) | | | | 42 (35-49) | | | 18 (12-26) | | 96 (90-99) | |
| SOC score | 12 (11-13) | 17 (11-23) | **0.0023** | 0.68 (0.57-0.79) | 9.472 | | | 89 (71-98) | | | | 30 (24-37) | | | 15 (10-22) | | 95 (86-99) | |
| EVMAP score, mean (CI) | 35 (34-36) | 40 (37-42) | **< 0.0001** | 0.75 (0.66-0.84) | 32.59 | | | 89 (71-98) | | | | 48 (41-56) | | | 20 (13-28) | | 97 (91-99) | |
| EVMAP + SOC score, mean (CI) | 11 (9-12) | 24 (16-32) | **< 0.0001** | 0.76 (0.67-0.86) | 7.332 | | | 89 (71-98) | | | | 49 (42-56) | | | 20 (13-28) | | 97 (91-99) | |
| DRE: Digital rectal exam | | | | | |  |  | |  |  |  | |  |  | |  | |  |
| PSA: Prostate specific antigen | | | | | |  |  | |  |  |  | |  |  | |  | |  |
| SOC: Standard of care features including PSA, age, race, DRE, previous negative biopsy, and PCa family history | | | | | |  |  | |  |  |  | |  |  | |  | |  |
| EVMAP: Extracellular vesicle machine learning analysis platform | | | | | |  |  | |  |  |  | |  |  | |  | |  |
| ROC AUC: Receiver operator characteristic area under the curve | | | | | |  |  | |  |  |  | |  |  | |  | |  |
| PPV: Positive predictive value | | | | | |  |  | |  |  |  | |  |  | |  | |  |
| NPV: Negative predictive value | | | | | |  |  | |  |  |  | |  |  | |  | |  |
